# Motivated Attention in Climate Change Perception and Action

**DOI:** 10.3389/fpsyg.2019.01541

**Published:** 2019-07-16

**Authors:** Yu Luo, Jiaying Zhao

**Affiliations:** ^1^Department of Psychology, University of British Columbia, Vancouver, BC, Canada; ^2^Institute for Resources, Environment and Sustainability, University of British Columbia, Vancouver, BC, Canada

**Keywords:** ideology, motivated reasoning, eyetracking, behavior change, climate communication

## Abstract

Despite the scientific consensus, some people still remain skeptical about climate change. In fact, there is a growing partisan divide over the last decade within the United States in the support for climate policies. Given the same climate evidence, why do some people become concerned while others remain unconvinced? Here we propose a motivated attention framework where socio-political motivations shape visual attention to climate evidence, altering perceptions of the evidence and subsequent actions to mitigate climate change. To seek support for this framework, we conducted three experiments. Participants viewed a graph of annual global temperature change while they were eyetracked and estimated the average change. We found that political orientation may bias attention to climate change evidence, altering the perception of the same evidence (Experiment 1). We further examined how attentional biases influence subsequent actions to mitigate climate change. We found that liberals were more likely to sign a climate petition or more willing to donate to an environmental organization than conservatives, and attention guides climate actions in different ways for liberals and conservatives (Experiment 2). To seek causal evidence, we biased attention to different parts of the temperature curve by coloring stronger climate evidence in red or weak climate evidence in red. We found that liberals were more likely to sign the petition or more willing to donate when stronger evidence was in red, but conservatives were less likely to act when stronger evidence was in red (Experiment 3). This suggests that drawing attention to motivationally consistent information increases actions in liberals, but discouraged conservatives. The findings provide initial preliminary evidence for the motivated attention framework, suggesting an attentional divide between liberals and conservatives in the perception of climate evidence. This divide might further reinforce prior beliefs about climate change, creating further polarization. The current study raises a possible attentional mechanism for ideologically motivated reasoning and its impact on basic perceptual processes. It also provides implications for the communication of climate science to different socio-political groups with the goal of mobilizing actions on climate change.

## Introduction

Climate change involves a significant change in weather patterns around the world due to increased concentrations of greenhouse gases in the atmosphere mostly driven by human activities over the last 50 years (Intergovernmental Panel on Climate Change, 2014). The atmospheric concentration of carbon dioxide has recently exceeded a mole fraction of 400 parts per million, higher than any century in the past 420,000 years (Petit et al., 1999; National Oceanic and Atmospheric Administration, 2018a,b). Because of the high concentration of CO_2_, global mean surface temperature increased by 0.87°C in the last decade compared to the average temperature from 1850 to 1900, and it is projected to reach 1.5°C between 2030 and 2052 (Intergovernmental Panel on Climate Change, 2018; National Oceanic and Atmospheric Administration, 2018a,b). The significant changes in CO_2_ concentration and global temperature pose a significant threat to humanity because of their severe, extensive, adverse impacts on human and natural systems. Studies have shown that climate change increases the frequency and severity of extreme weather events (Intergovernmental Panel on Climate Change, 2015; Wuebbles et al., 2017), impairs global food production (Lobell et al., 2008), shrinks ice volume and snow cover (Robinson et al., 2014), causes sea levels to rise (Kniveton, 2017; Nerem et al., 2018), and leads to forest disturbances (Dale et al., 2001; Seidl et al., 2017) and deterioration of terrestrial and marine ecosystems (Rosenzweig et al., 2008; Hoegh-Guldberg and Bruno, 2010; Levitus et al., 2017). In fact, the combined value of damages across agriculture, coastal storms, energy, human mortality, and labor sectors costs roughly 1.2% of gross domestic product per +1°C on average in the United States (Hsiang et al., 2017).

The scientific evidence for climate change has been unequivocal (Intergovernmental Panel on Climate Change, 2014). In fact, 97% of actively publishing climate scientists agree that human activities are causing global warming (Cook et al., 2013, 2016). However, some people still remain skeptical about climate change despite the scientific consensus (e.g., Hulme, 2009; Poortinga et al., 2011; Weber and Stern, 2011; Hornsey et al., 2016). Within the United States, public views on climate change tend to polarize along party lines (McCright and Dunlap, 2011). According to a recent Gallup Poll, 86% of democrats vs. 42% of republicans agree most scientists believe global warming is occurring; 4% of democrats vs. 69% of republicans think the seriousness of global warming is generally exaggerated; 89% of democrats vs. 35% of republicans believe global warming is caused by human activities; and 91% of democrats vs. 33% of republicans worry about global warming (Brenan and Saad, 2018). This partisan divide has not only endured, but widened over time. A poll from Pew Research Center shows that in 2006, 79% of democrats vs. 59% of republicans said there is solid evidence that the average temperature on Earth has been getting warmer, but in 2017, 92% of democrats vs. 52% of republicans said so (Pew Research Center, 2017). There is also a growing divide in policy priorities: in 1994, 66% of democrats vs. 58% of republicans said stricter environmental laws and regulations are worth the cost, but in 2017, 77% of democrats vs. 36% of republicans said so (Pew Research Center, 2017); in 2008, 47% of democrats vs. 15% of republicans said climate change is a top priority for the president and congress, but in 2018, 68% of democrats vs. 18% of republicans said so (Pew Research Center, 2018).

To explain public skepticism on climate change, traditional accounts have adopted an information deficit model that attributes disbelief to a lack of knowledge or understanding (Lorenzoni et al., 2007; Shi et al., 2016), a lack of affect (Leiserowitz, 2006), or insufficient awareness about the issue (Norton and Leaman, 2004; Lorenzoni and Pidgeon, 2006). However, these accounts have failed to explain the partisan polarization over the years when an increasing volume of information and evidence on climate change has been presented to the public. Another conundrum is that individuals with high science literacy and technical reasoning skills are not the most concerned about climate change, but rather, they are the ones among whom polarization is the greatest (Kahan, 2012; Drummond and Fischhoff, 2017; Kahan et al., 2017). This suggests that the public divide on climate change is not solely driven by a lack of understanding or knowledge, and the mere presentation of climate change evidence is likely insufficient to convince the public.

Recent efforts to explain group polarization have relied on a motivated reasoning approach that traces back to studies on motivated social cognition in the early 1950’s. In a pioneering study, Hastorf and Cantril (1954) demonstrated that after watching the same football game between Princeton and Dartmouth teams, Princeton and Dartmouth students drew distinct conclusions about the game, where they largely disagreed on the number of infractions made by each team, the reasons behind these infractions, the roughness of the game, and who started the rough play. This study suggests that the same sensory input is interpreted in vastly different ways depending on the viewer’s social affiliations, predispositions, and motivations.

Following the same logic, recent theories focus on identity-based polarization, where perceptions of controversial topics such as climate change are driven by socio-political motivations and beliefs (Drummond and Fischhoff, 2017; Kahan et al., 2017; Bail et al., 2018; Ehret et al., 2018). Specifically, the cultural cognition thesis posits that people form perceptions of risks or controversial topics in a way that coheres with values characteristic of the groups with which they identify (Kahan et al., 2011; Kahan, 2012). One explanation underlying this thesis is that people selectively expose themselves to information from news media that is consistent with their existing motivations and beliefs (Feldman et al., 2012; Newman et al., 2018). Similarly, the identity-protective cognition thesis argues that people high on numeracy skills use their quantitative-reasoning capacity to selectively interpret the data to conform to their cultural and political values (Kahan et al., 2017). Another account suggests that people automatically obey in-group norms and oppose out-group norms, but critically, they exaggerate the extent of opposition from out-group members, creating a false sense of cultural norm on climate change (Van Boven et al., 2018).

**Figure 1:**
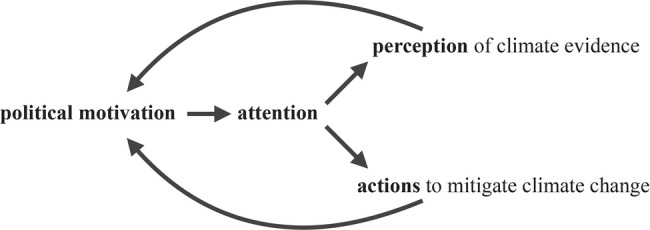
A motivated attention framework of climate change perception and action.

Although many studies have suggested social motivations and ideologies determine the interpretation of quantitative evidence, it is currently unknown *how* motivations and ideologies shape perception and judgment. To specifically examine how this process occurs, here we propose a motivated attention framework to offer an attentional mechanism to explain the political polarization on climate change. Specifically, socio-political motivations shape visual attention to climate change evidence, altering the perception of the evidence and subsequent actions to mitigate climate change. The altered perception and actions can further reinforce prior beliefs and motivations, thus creating a positive feedback loop (Figure 1). This framework is supported by our previous work that demonstrates that liberals who are concerned about climate change attend more readily to climate-related words over neutral words, but conservatives who are not concerned about climate change do not show an attentional priority of climate-related words over neutral words, suggesting that political orientations are associated with different attentional priorities of climate change (Whitman et al., 2018).

In the motivated attention framework, we define political motivation as political orientation, and we predict that liberals and conservatives attend to the same climate change evidence (i.e., a graph of global temperature) in ways that are consistent with their political norms. Attention is measured by eye gaze dwell time on the graph. We define perception of climate evidence as the estimation of global temperature from the graph, and actions to mitigate climate change as the likelihood to sign climate petitions or donate to an environmental organization. To seek evidence for this framework, we conducted three experiments to examine how people with different political orientations perceive the same global temperature graph and whether the perceptual differences can be explained by different attentional priorities (Experiment 1), how these attentional biases alter actions to mitigate climate change (Experiment 2), and how drawing attention to motivationally consistent evidence influences climate actions (Experiment 3).

## Experiment 1

This experiment examines how political motivation alters the perception of climate change evidence. We predict that people with different political orientations perceive the same temperature graph differently when the graph is framed as global temperature, but not when the graph is under a neutral frame (i.e., when the evidence is not motivationally relevant). We further examine whether the perceptual differences can be explained by different attentional allocations on the graph. We tracked visual attention using an eyetracker in the lab while participants were viewing the graph. We predict that liberals and conservatives focus on different parts of the graph consistent with their political motivations to guide their temperature estimation.

### Participants

A total of 213 undergraduate students (142 females; mean age = 20.3 years, SD = 2.7) from University of British Columbia (UBC) participated for course credit. Six participants who provided an estimation above or below 2.5 standard deviations of the group mean were excluded from the study, leaving a final sample of 207. All three experiments reported here were approved by UBC Behavioral Research Ethics Board. All participants in the experiments provided informed consent.

### Stimuli

**Figure 2:**
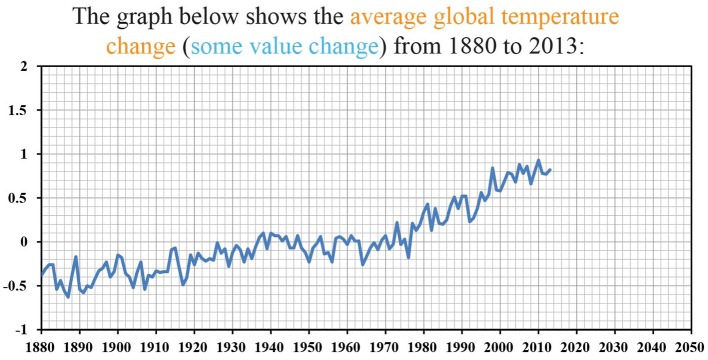
A graph of global annual mean surface air temperature change in Celsius (°C) from 1880 to 2013 in the temperature condition, or neutral value change in the neutral condition.

We used a graph of global annual mean surface air temperature change in Celsius (°C) from 1880 to 2013 (Figure 2), generated from estimates based on land data only^1^ provided by National Aeronautics and Space Administration (NASA). The y-axis was the temperature change relative to a baseline period from 1951 to 1980, a reference period used by NASA. Specifically, the temperature change was the difference between the global mean surface air temperature in each year and the mean temperature from 1951 to 1980 (baseline period). The graph subtended 27.2° of visual angle in width (916 pixels) and 15.9° of visual angle in height (527 pixels).

### Procedure

Participants were randomly assigned to one of two conditions: temperature condition (*N* = 104) or neutral condition (*N* = 103). In the temperature condition, participants viewed the graph of annual global temperature change. In a descriptive paragraph above the graph, participants were informed that the graph showed the annual global temperature change from 1880 to 2013. There was no label for the x-axis or the y-axis. Participants were eyetracked when they were viewing the graph. Eye gaze was tracked using an SMI RED-250 Mobile Eyetracking System (60 Hz). Each participant was seated 50 cm from a computer monitor with a resolution of 1920 pixels × 1080 pixels. After seeing the graph, participants were asked to estimate the average global temperature change from 1880 to 2013. In the neutral condition, participants viewed the exact same graph but without any framing related to global temperature, and the x-axis and the y-axis were exactly same as in the temperature condition. In the descriptive paragraph above the graph, participants were informed that the graph showed some value change from 1880 to 2013. Same as in the temperature condition, participants were eyetracked when they were viewing the graph. After seeing the graph, participants were asked to estimate the average value change from 1880 to 2013. After the estimation task, participants in both conditions provided their demographic information and rated their political orientation on an 11-point scale from −5 (very liberal, left-wing) to 5 (very conservative, right-wing). In our analysis, we divided participants into a liberal group (whose ratings on the political orientation scale were below 0) and a conservative group (whose ratings on the political orientation scale were above 0). In the temperature condition, the mean rating on the political orientation scale was −1.36 (69 liberals and 21 conservatives). In the neutral condition, the mean rating on the political orientation scale was −1.57 (69 liberals and 21 conservatives).

### Results and Discussion

#### Estimation Results

The objective average change in the graph was 0.01, and participants in both conditions over-estimated the average value change (mean estimated change = 0.52 in the temperature condition, mean estimated change = 0.43 in the neutral condition, *p* < 0.001). Participants in the temperature condition estimated the change as numerically larger than those in the neutral condition [*t*(205) = 1.49, *p* = 0.14, *d* = 0.20].

The goal of this experiment was to examine how people with different political orientations perceive the same global temperature graph. In the temperature condition, we found that more liberalism was weakly correlated with higher estimates of the temperature change [*r*(102) = −0.19, *p* = 0.055], suggesting liberals tended to perceive a higher temperature change than conservatives. However in the neutral condition, no correlation was found between political orientation and estimation of temperature change [*r*(101) = 0.02, *p* = 0.83]. These results suggest that political orientation is associated with different perceptions of the same evidence when the graph is framed as global temperature, but not when the graph is under a neutral frame (i.e., when the evidence is not motivationally relevant).

#### Eyetracking Results

**Figure 3:**
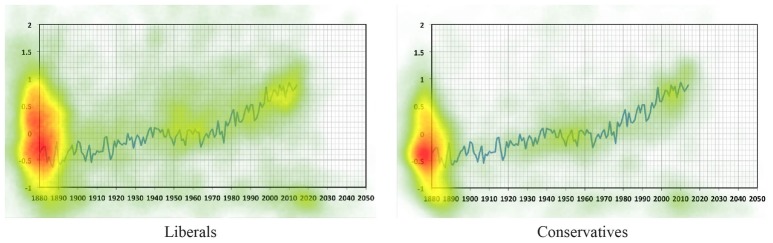
A heatmap showing the average duration of dwell time on the temperature curve for liberals (left, *N* = 69) and conservatives (right, *N* = 21) in the temperature condition. Participants whose ratings on the political orientation scale were below 0 were grouped as liberals and whose ratings on the political orientation scale were above 0 were grouped as conservatives. Warmer colors represent higher average duration of dwell time.

The heatmaps of the average dwell time for liberals and conservatives in the temperature condition are shown in Figure 3. The heatmap of the average dwell time for independents (whose ratings on the political orientation scale were 0) in the temperature condition is shown in Section A of Supplementary Materials.

From Figure 3, it is evident that both liberals and conservatives looked more at the relatively flat phase of the curve from 1940 to 1980 and the rising phase of the curve from 1990 to 2013. We therefore defined two areas of interest (AOIs) on the curve: the flat phase (1940 to 1980, subtending 5.7° of visual angle in width, 187 pixels, and 1.8° in height, 59 pixels) which we interpret as weaker evidence of climate change, and the rising phase (1990 to 2013, subtending 3.4° of visual angle in width, 112 pixels, and 3.0° in height, 98 pixels) which we interpret as stronger evidence of climate change^2^. To measure visual attention, we calculated dwell time in each AOI. Since participants spent different amounts of time on the graph, we calculated the proportional gaze dwell time for each participant, which was defined as the dwell time spent in each AOI divided by the total dwell time on the graph.

To examine the overall relationship between participants’ political motivation and their visual attention on the graph, we correlated political orientation and the difference in proportional dwell time between the rising phase and the flat phase (rising − flat). There was no significant correlation between political orientation and the difference in proportional dwell time between the rising phase and the flat phase in the temperature condition [*r*(102) = −0.15, *p* = 0.13] or the neutral condition [*r*(101) = −0.02, *p* = 0.86]. However, relative to the neutral condition, in the temperature condition, more liberalism tended to be associated with greater proportional dwell time on the rising phase relative to the flat phase. These results point to a possibility that the more liberal the participants, the more attention they paid to the rising phase relative to the flat phase of the temperature curve. We further divided the participants into a liberal group and a conservative group. In the temperature condition, we did not find any correlation between attention on the graph and the degree of liberalism [*r*(67) = −0.14, *p* = 0.26] or conservatism [*r*(19) = 0.08, *p* = 0.74]. In the neutral condition, we did not find any correlation between attention on the graph and the degree of liberalism [*r*(69) = −0.05, *p* = 0.70] or conservatism [*r*(19) = −0.32, *p* = 0.17].

We then examined the relationship between visual attention and estimation. In the temperature condition, proportional dwell time on the rising phase relative to the flat phase was positively correlated with estimates of temperature change [*r*(102) = 0.33, *p* < 0.001], but not in neutral condition [*r*(101) = 0.05, *p* = 0.60]. This suggests that more attention to the rising phase relative to the flat phase of the curve (i.e., more attention to stronger evidence of climate change) was associated with higher estimations of temperature change. We further divided the participants into a liberal group and a conservative group. In the temperature condition, we found that liberals who focused more on the rising phase of the curve relative to the flat phase provided higher estimates of temperature change [*r*(67) = 0.30, *p* = 0.01], and for conservatives, there was a marginal correlation [*r*(19) = 0.38, *p* = 0.09]. We note that although the value of *p* was marginal, the correlation coefficient was larger for conservatives than for liberals. This may be due to the smaller sample size of conservatives. The correlation coefficient may change if the sample size of conservatives increased. In the neutral condition, no correlation was found for liberals [*r*(67) = 0.09, *p* = 0.46] or conservatives [*r*(19) = −0.01, *p* = 0.95]. In sum, these results provide initial evidence suggesting that political orientation could be associated with attentional biases which alter the perception of the same evidence.

## Experiment 2

Experiment 1 involved a sample of undergraduate students, therefore limiting the generalizability of the findings. Experiment 2 aimed to replicate Experiment 1 with a broader online sample on Amazon Mechanical Turk (MTurk) using a novel attention-tracking technique called BubbleView. More importantly, this experiment aimed to examine how attentional biases were related to actions to mitigate climate change.

### Participants

A new group of 180 participants (58 females; mean age = 38.0 years, SD = 11.9) were recruited on MTurk. All participants gave informed consent and received US$0.25 each as compensation for participation. All participants were from the United States. Three participants who provided an estimation above or below 2.5 standard deviations of the group mean were excluded from the study, leaving a final sample of 177.

### Stimuli and Procedure

**Figure 4:**
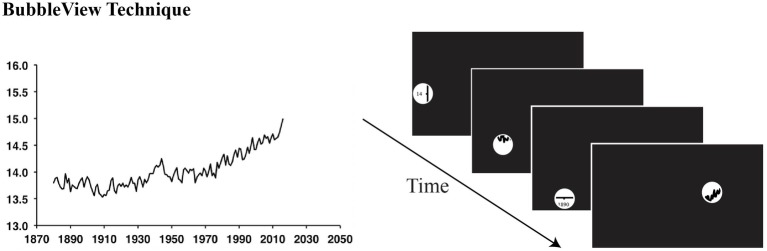
Experiment 2 Methods. Using the BubbleView technique, the graph on the left was covered by a black mask, and only a small circular area around the mouse was transparent. Participants had to move their mouse to see the graph.

The stimulus in this experiment was a graph showing annual global temperature, rather than temperature change as in the previous experiment. This was to ease the estimation of global temperature, as estimating average temperature change may be more difficult than estimating average temperature. Specifically, the graph showed the global annual mean surface air temperature in Celsius (°C) from 1880 to 2017 (Figure 4), generated from estimates based on land and ocean data^3^ provided by NASA. The graph was changed from land only to land and ocean to show a more representative view of the annual global temperature over years. The graph subtended 23.4° of visual angle in width (783 pixels) and 13.6° in height (450 pixels), assuming that participants were seated 50 cm from a computer monitor with a resolution of 1920 pixels × 1080 pixels.

As in the previous experiment, participants were randomly assigned to the temperature condition (*N* = 87) or the neutral condition (*N* = 90). To measure participants’ attention, we used a novel online attention-tracking tool called BubbleView (adapted from Kim et al., 2017). The entire graph, including the x-axis and the y-axis, was covered by a black mask, and only a small circular area around the mouse was transparent where the participant could see the underlying graph (see Figure 4). Participants were asked to move their mouse to see the content of the graph. The black mask was the same size as the graph. The transparent circular area subtended 1.2° of visual angle (diameter = 40 pixels). We tracked participants’ mouse location as a proxy for visual attention.

After viewing the graph, participants were asked to estimate the average global temperature (°C) in the temperature condition, or the average value in the neutral condition from 1880 to 2017. They were asked to provide an estimate between 13 and 16 (the bounds of the y-axis). After providing the estimates, participants were presented with a petition to stand with the Nature Conservancy to call on United States leaders to stand strong on climate change, and were asked whether they were willing to sign it. Participants also indicated whether they were willing to donate to Natural Resource Defense Council. The order of the petition and the donation question was random. If participants were willing to sign the pledge, they provided their name and email which were then forwarded to change.org. If participants were willing to donate, they were asked to indicate the amount of donation (which was only hypothetical). At the end of the experiment, participants in both conditions provided their demographic information and rated their political orientation on the same 11-point scale. As in the previous experiment, we divided participants into a liberal group and a conservative group. In the temperature condition, the mean rating on the political orientation scale was −0.82 (47 liberals and 29 conservatives). In the neutral condition, the mean rating on the political orientation scale was 0.11 (32 liberals and 39 conservatives).

### Results and Discussion

#### BubbleView Results

This experiment aimed to replicate the attentional results from Experiment 1 using the BubbleView technique. To measure attention, we calculated the number of mouse locations in each AOI (defined below). Since there was no time limit for participants to view the graph, we used the proportional number of mouse location, which was calculated as the number of mouse locations in each AOI divided by the total number of mouse locations on the graph.

Similar to Experiment 1, we defined two AOIs on the temperature curve, one on the flat phase from 1880 to 1948 (subtending 8.3° of visual angle in width, 274 pixels, and 3.6° in height, 120 pixels), another on the rising phase from 1949 to 2017 (subtending 7.7° of visual angle in width, 254 pixels, and 5.2° in height, 170 pixels). We adjusted the AOIs from Experiment 1 because this graph was slightly different from the graph in Experiment 1, as it contained both land and ocean data, whereas the one in Experiment 1 contained only land data. This means that there was a small peak of global temperature around 1945 in the current graph, whereas the curve in Experiment 1 was relatively flat before 1980. For this reason, we tried to divide the curve into two halves (with the year 1949 being the mid-point), and the second half of the temperature curve was defined as the rising phase, which was steeper than the first half of the curve defined as the flat phase.

We first examined the relationship between political orientation and attention. This time, we did not find a correlation between political orientation and the proportional number of mouse location in the rising phase relative to the flat phase in the temperature condition [*r*(85) = 0.07, *p* = 0.49] or in the neutral condition [*r*(88) = −0.03, *p* = 0.81]. This did not replicate the numerical trend in Experiment 1. We further divided the participants into a liberal group and a conservative group. In the temperature condition, same as in Experiment 1, we did not find any correlation between attention on the graph and the degree of liberalism [*r*(45) = 0.12, *p* = 0.44] or conservatism [*r*(27) = 0.09, *p* = 0.64]. In the neutral condition, we did not find any correlation between attention on the graph and the degree of liberalism [*r*(30) = −0.10, *p* = 0.58] or conservatism [*r*(37) = 0.20, *p* = 0.22].

We then examined the relationship between attention and temperature estimation. In the temperature condition, we found that greater proportional number of mouse location in the rising phase relative to the flat phase was positively correlated with higher estimate of temperature [*r*(85) = 0.34, *p* = 0.001], but no correlation was found in the neutral condition [*r*(88) = −0.10, *p* = 0.37]. This result replicated the findings in Experiment 2, suggesting that more attention to the rising phase relative to the flat phase of the temperature curve (i.e., more salient evidence of climate change) was associated with higher estimations of global temperature.

**Figure 5:**
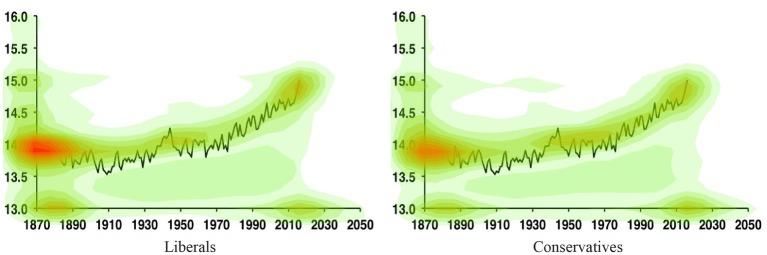
A heatmap showing the distribution of the average density of mouse location on the graph for liberals (left, *N* = 47) and for conservatives (right, *N* = 29) in the temperature condition. Participants whose ratings on the political orientation scale were below 0 were grouped as liberals and whose ratings on the political orientation scale were above 0 were grouped as conservatives. Warmer colors represent higher average density of mouse location.

The heatmaps of the average density of mouse location on the graph for liberals and conservatives are shown in Figure 5. The heatmap of the average dwell time for independents (whose ratings on the political orientation scale were 0) in the temperature condition is shown in Section B of Supplementary Materials. In the temperature condition, liberals who focused more on the rising phase relative to the flat phase provided marginally higher estimates of temperature [*r*(45) = 0.28, *p* = 0.054], and the same marginal correlation was found for conservatives [*r*(27) = 0.35, *p* = 0.06]. In the neutral condition, no correlation was found for liberals [*r*(30) = 0.10, *p* = 0.59] or conservatives [*r*(37) = −0.25, *p* = 0.13]. These results suggest that both liberals and conservatives who focused more on the rising phase relative to the flat phase tended to provide a higher estimation of global temperature, which partially replicated Experiment 1 in which we found that liberals who focused more on the rising phase showed significantly higher perceived temperature, but this correlation was only marginal for conservatives.

#### Climate Action Results

A more important goal of this experiment was to examine how attentional biases were related to actions to mitigate climate change. We first conducted log linear analyses on petition signing in the three-way contingency table. We found a significant three-way interaction [*G*^2^(4) = 21.50, *p* < 0.001]. We then conducted separate chi-square tests for the temperature condition and the neutral condition. We found that more liberals signed the climate-related petition than conservatives did in the temperature condition (*X*^2^ = 10.19, *p* = 0.001), but not in the neutral condition (*X*^2^ = 1.74, *p* = 0.19) (Table 1). This suggests that when the evidence was motivationally relevant, people were more likely to behave in ways that were consistent with their political orientations.

**Table 1:** The number of liberals and conservatives who signed the petition or were willing to donate in the temperature condition and the neutral condition.

Condition	PO	Yes	No	Percent Yes	Chi-square
**Petition signing**			
Temperature	Liberals	25	22	53.2%	*X*^2^ = 10.19, *p* = 0.001
	Conservatives	4	25	13.7%	
Neutral	Liberals	12	20	37.5%	*X*^2^ = 1.74, *p* = 0.19
	Conservatives	8	31	20.5%	
**Donation willingness**			
Temperature	Liberals	16	31	34.0%	*X*^2^ = 0.97, *p* = 0.32
	Conservatives	6	23	20.7%	
Neutral	Liberals	9	23	28.1%	*X*^2^ = 2.65, *p* = 0.10
	Conservatives	4	35	10.3%	

For willingness to donate, we found that there was a significant three-way interaction [*G*^2^(4) = 12.37, *p* = 0.02]. However, we did not find a difference in the willingness to donate between liberals and conservatives in the temperature condition (*X*^2^ = 0.97, *p* = 0.32) or in the neutral condition (*X*^2^ = 2.65, *p* = 0.10) (Table 1). The null results in donation could be driven by the fact that the donation question was hypothetical and no actual donations were made.

#### Climate Action and Attention Results

In the final analysis, we examined the relationship between attention and the likelihood to sign the petition (we did not consider the donation results as they were insignificant in the previous section). In the temperature condition, greater attention to the flat phase relative to the rising phase was associated with a higher likelihood of signing the petition for liberals [*r*(45) = −0.32, *p* = 0.03]. However, greater attention to the rising phase relative to the flat phase was marginally associated with a higher likelihood of signing the petition for conservatives [*r*(27) = 0.33, *p* = 0.08]. In the neutral condition, there was no correlation between attention and willingness to sign for liberals [*r*(30) = 0.21, *p* = 0.25] or conservatives [*r*(37) = −0.23, *p* = 0.15]. This suggests that attention guides climate actions in different ways for liberals and conservatives.

## Experiment 3

The previous two experiments were correlational by nature. To seek causal evidence for the motivated attention framework, we manipulated attention by coloring different parts of the temperature curve to deliberately bias attention to stronger or weaker evidence of climate change. In other words, we aimed to examine how drawing attention to motivationally consistent evidence influences subsequent actions.

### Participants

A new group of 278 participants (155 females; mean age = 37.5 years, SD = 13.0) was recruited from MTurk. All participants gave informed consent and received US$0.25 for participating. All participants were from the United States.

### Stimuli and Procedure

**Figure 6:**
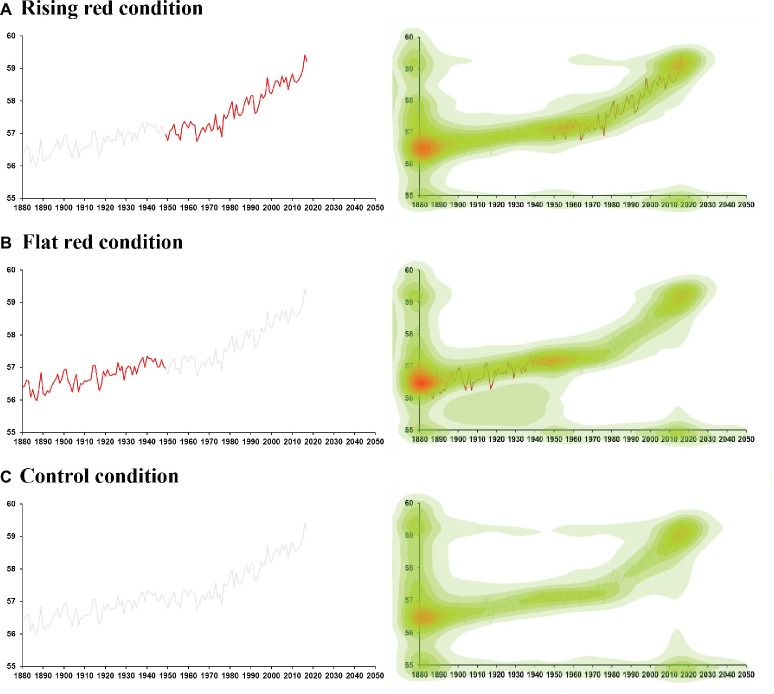
Experiment 3. **(A)** In the rising red condition, the rising phase from 1950 to 2017 was highlighted in red. **(B)** In the flat red condition, the flat phase from 1880 to 1949 was highlighted in red. **(C)** In the control condition, both rising and flat phases were in gray. A heatmap representing the distribution of the average density of mouse location on the graph is shown on the right side in each condition. Warmer colors represent higher average density of mouse location.

Given the slight increase in temperature from 1930 to 1945 on the land and ocean graph in Experiment 2 may diminish any attentional bias to the rising phase, the stimulus used in the current experiment was the same global temperature graph used in Experiment 1, generated from estimates based on land data only^4^ provided by NASA, except that we converted the unit of global temperature from Celsius to Fahrenheit to facilitate the comprehension of temperature for United States participants, and we also updated the graph from 2013 to 2017. To manipulate attention, we highlighted the rising phase from 1950 to 2017 in red in the “rising red” condition (*N* = 105, Figure 6A), or highlighted the flat phase from 1880 to 1949 in red in the “flat red” condition (*N* = 84, Figure 6B), or did not highlight the curve in the control condition (*N* = 89, Figure 6C). In the rising red condition, the mean rating on the political orientation scale was −1.10 (60 liberals and 26 conservatives). In the flat red condition, the mean rating on the political orientation scale was −1.23 (42 liberals and 25 conservatives). Attention was again tracked using BubbleView as in Experiment 2. After viewing the graph, participants were asked whether they were willing to sign a climate-related petition and whether they were willing to donate to an environmental organization, as in Experiment 2.

### Results and Discussion

#### Manipulation Check

As a manipulation check, we examined attention allocation in different phases of the curve in each condition. Participants in the rising red condition paid more attention to the rising phase (AOI fixation = 28.6%) compared to that in the flat red condition (AOI fixation = 25.3%) and control condition (AOI fixation = 24.6%) [*F*(2,275) = 4.86, *p=* 0.008, $\eta_{p}^{2}$ = 0.03]. Participants in the flat red condition paid marginally more attention to the flat phase (AOI fixation = 23.4%) compared to that in the rising red condition (AOI fixation = 22.3%) and control condition (AOI fixation = 20.2%) [*F*(2,275) = 2.65, *p =* 0.07, $\eta_{p}^{2}$ = 0.03]. This suggests our manipulation of attention was successful, specifically, highlighting the rising phase in red drew more attention to the rising phase, and highlighting the flat phase in red drew more attention to the flat phase. The heatmaps of the average density of mouse location on the graph in each condition are shown in Figure 6.

#### Climate Action Results

We first conducted log linear analyses on petition signing in the three-way contingency table. We found a significant three-way interaction among condition (rising vs. flat), political orientation (liberals vs. conservatives), and signing (yes vs. no) [*G*^2^(4) = 13.31, *p* = 0.01]. This interaction suggests that more liberals than conservatives signed the petition when the rising phase was highlighted, but not when the flat phase was highlighted. To probe this interaction further, we conducted separate chi-square tests for the rising red condition and the flat red condition. Liberals were more likely to sign the petition than conservatives when the rising phase was highlighted (*X*^2^ = 8.80, *p* = 0.003). When the flat phase was highlighted, there was no significant difference between liberals and conservatives (*X*^2^ = 0.66, *p* = 0.42) (Table 2). This suggests that liberals were more likely to sign the petition when the rising phase was highlighted than when the flat phase was highlighted.

**Table 2:** The number of liberals and conservatives who signed the petition or were willing to donate in the rising red and the flat red conditions.

Condition	PO	Yes	No	Percent Yes	Chi-square
**Petition signing**			
Rising red	Liberals	34	26	56.7%	*X*^2^ = 8.80, *p* = 0.003
	Conservatives	5	21	19.2%	
Flat red	Liberals	19	23	45.2%	*X*^2^ = 0.66, *p* = 0.42
	Conservatives	8	17	32.0%	
**Donation willingness**			
Rising red	Liberals	31	29	51.7%	*X*^2^ = 13.03, *p* < 0.001
	Conservatives	2	24	7.7%	
Flat red	Liberals	15	27	35.7%	*X*^2^ = 0.53, *p* = 0.47
	Conservatives	6	19	24.0%	

For willingness to donate, we again found a significant three-way interaction [*G*^2^(4) = 20.00, *p* < 0.001]. Moreover, liberals were more willing to donate than conservatives when the rising phase was highlighted (*X*^2^ = 13.03, *p* < 0.001), but there was no significant difference between liberals and conservatives when the flat phase was highlighted (*X*^2^ = 0.53, *p* = 0.47). This again suggests that liberals were more willing to donate when the rising phase was highlighted than when the flat phase was highlighted.

The results collectively suggest that drawing attention to more salient evidence of climate change encouraged actions in liberals. Although there was no significant difference between the two groups when the flat phase was highlighted, there was a numerical increase in both petition signing and donation willingness for conservatives when the flat phase was highlighted than when the rising phase was highlighted. In sum, these results provide initial evidence that drawing attention to motivationally consistent evidence can increase actions.

## General Discussion

The goal of the current study was to examine how political motivations (i.e., political orientation) shape visual attention to climate information and how these attentional biases alter the perception of climate evidence and influence subsequent actions to mitigate climate change. We propose a motivated attention framework that offers a cognitive pathway underlying the partisan divide on climate change (Figure 1). Specifically, the framework suggests that socio-political motivations shape attention to climate information, altering perception of climate evidence and subsequent actions.

In three experiments, we provided initial preliminary evidence to support the motivated attention framework. In Experiment 1, we found that liberals tended to attend more to the rising phase of the temperature curve than the flat phase of the curve and give a higher estimate of global temperature change than conservatives did. In addition, liberals who attended more to the rising phase of the curve relative to the flat phase gave a higher estimate of temperature change, but this effect was weaker in conservatives. However, this result was not found in the neutral condition. Thus, attending more to the rising phase of the temperature curve may induce concerns in liberals, leading to a bias in their estimation of the global temperature change, but attending more to the rising phase of the neutral curve does not alter the perception of the evidence because of a lack of prior motivations. These results suggest that political orientation can bias visual attention to climate change evidence, which alters the perception of the evidence.

In Experiment 2, we partially replicated the findings in Experiment 1 with a larger sample in the United States using the BubbleView technique to track attention online. The lack of a correlation between political orientation and attention on the rising phase in Experiment 2 could be driven by the fact that the flat phase was indeed less flat than that in Experiment 1. Since the global temperature graph contained both land and ocean data, there was a slight increase from 1930 to 1945. This increase may have made the flat phase and the rising phase more similar, therefore diminishing any attentional bias to the rising phase.

A more important finding in Experiment 2 was that the attentional difference between the rising phase and the flat phase was negatively correlated with the likelihood of signing the petition for liberals, but positively correlated for conservatives. One explanation is that liberals were equally sensitive to the increase in global temperature from 1930 to 1945 in the flat phase and from 1949 to 2017 in the rising phase, so focusing on both phases equally was associated with a higher likelihood of signing the petition. Another explanation is that liberals who were inherently more likely to sign the petition attended to the increases in temperature in both phases equally. For conservatives, however, the relationship was reversed. There were two possible interpretations of the correlation: those who attended more to the rising phase than the flat phase became more concerned with climate change and therefore were more likely to sign the petition; or, those who are more likely to sign a petition generally attend to the rising temperature as evidence for their action. Since the relationship was only correlational, we cannot identify the directionality. Nonetheless, the results from Experiment 2 suggest that attention guides climate actions in different ways for liberals and conservatives.

Experiment 3 examined the causality between attention and climate action by drawing attention to the rising phase or the flat phase. We found that liberals were more likely to sign the petition or donate to an environmental organization when the rising phase (their motivationally consistent evidence) was highlighted. However, conservatives were more likely to sign or donate when the flat phase (their motivationally consistent evidence) was highlighted. These results suggest that drawing attention to motivationally consistent evidence increases actions for both liberals and conservatives. Critically, the evidence is different for liberals and conservatives depending on their motivations. This also suggests that the same approach that works for liberals may not work for conservatives.

An important limitation of the current study is that it does not provide evidence on how altered perceptions and actions reinforce prior motivations. This is a promising avenue for future studies that can examine this positive feedback loop between actions and motivations. For interventions, future studies can also investigate ways to break the feedback loop to prevent further polarization. For example, since conservatives were more likely to act when they voluntarily attended to stronger evidence of climate change, but not when their attention was deliberately drawn to the evidence, one solution is to implicitly bias conservatives’ visual attention to stronger climate change evidence, such as framing the evidence in a way that is consistent with their values and beliefs (Bain et al., 2012). In the current study, we tested people’s civic actions in the public sphere by asking them to sign a petition and donate to an environmental organization. Future studies can generalize the findings to behaviors in the private sphere, such as how likely they are to drive or fly less.

The current study suggests that ideologically driven motivations can influence basic perceptual processes. It reveals an attentional pathway underlying motivated reasoning, which helps explain group polarization. For example, students from different schools may attend to different players in the same game, which led to different perceptions of the game (Hastorf and Cantril, 1954). Liberals and conservatives may attend to different numbers in the same table, which led to different perceptions of risk (Kahan et al., 2011). People with greater science literacy or high numeracy skills may be better able to selectively attend to different sources of evidence, which can lead to greater group polarization (Drummond and Fischhoff, 2017; Kahan et al., 2017).

Beyond group polarization, the current study provides an attentional account to several well-established phenomena in social psychology. For example, cognitive dissonance is triggered when the evidence presented is inconsistent with a person’s beliefs, which can motivate the person to try to reduce dissonance (Festinger, 1962). One way to reduce dissonance is to avoid focusing attention on situations or information which will likely increase dissonance, and pay greater attention to information which will help to achieve consonance. Another example is confirmation bias where a person seeks or interprets evidence in ways that confirm existing beliefs, expectations, or a hypothesis in mind (Nickerson, 1998). The person may increase his/her attention to evidence that confirms their prior beliefs and suppress attention to evidence that disconfirms their beliefs. A third example is the central and peripheral route to persuasion, where the former involves a deliberate analysis of the content of the message, and the latter uses simple cues in the context (Petty and Cacioppo, 1986). The central route is employed when a person is motivated and has the ability to process the arguments in the message. Otherwise, the peripheral route takes place. When the evidence is consistent with people’s motivations, they may pay more attention to the evidence to deliberately analyze the information, which follows the central route. When the evidence is inconsistent with their motivations, they may pay less attention to the evidence and instead to the peripheral cues, which follows the peripheral route. As shown in one of the past studies, when a message is framed consistently with one’s value, it is more likely to be processed deliberately, and the strength of the argument influenced one’s attitude (von Borgstede et al., 2014).

In addition to theoretical implications, the current findings have potential practical implications for climate communication that uses data visualization to engage the public and policymakers (Harold et al., 2016; Bosetti et al., 2017; Zhao, 2017). First, the current study suggests that providing climate change evidence alone is likely to be insufficient since people may pay attention differently depending on their motivations. Second, the study suggests that we cannot use the same communication strategy for liberals and conservatives. For example, in Experiment 3, we found that drawing attention to more convincing evidence of climate change encouraged more liberals to act, but discouraged conservatives. Third, climate communication needs to align with ideological motivations to capture people’s attention. One approach is to frame climate change consistently with people’s values, such as framing mitigation efforts as promoting a warmer society and economic or technological development (Bain et al., 2012). Another approach is to provide information on peer group norms to shift attention, since people may have incorrect beliefs of how their peers view a controversial issue (Van Boven et al., 2018).

The current study is significant in several ways. First, it provides an attentional mechanism to understand group polarization on climate change. Specifically, our results provide initial preliminary evidence for the motivated attention framework, suggesting an attentional divide between liberals and conservatives. Second, the current study has implications for theories of ideologically motivated reasoning, demonstrating their influence on basic perceptual processes. Third, we offer a free new tool, BubbleView, to track attention online, which is more cost-effective compared to a conventional eyetracker. Finally, our findings have implications for climate communication and the design of behavioral interventions to mobilize actions on climate change in different socio-political groups.

## Ethics Statement

This study was carried out in accordance with the recommendations of the University of British Columbia Behavioral Research Ethics Board with written informed consent from all subjects. All subjects gave written informed consent in accordance with the Declaration of Helsinki. The protocol was approved by the University of British Columbia Behavioral Research Ethics Board.

## Author Contributions

YL and JZ contributed to the conception and design of the study. YL organized the database and analyzed and interpreted the data under the supervision of JZ. Both authors wrote the first draft of the manuscript, contributed to manuscript revision, read and approved the submitted version.

### Conflict of Interest Statement

The authors declare that the research was conducted in the absence of any commercial or financial relationships that could be construed as a potential conflict of interest.

## Footnotes

^1^Data retrieved from: https://data.giss.nasa.gov/gistemp/graphs/graph_data/Global_Mean_Estimates_based_on_Land_Data_only/graph.csv

^2^We define “flat” and “rising” in a relative sense given their different steepness.

^3^Data retrieved from: https://data.giss.nasa.gov/gistemp/graphs/graph_data/Global_Mean_Estimates_based_on_Land_and_Ocean_Data/graph.csv

^4^Data retrieved from: https://data.giss.nasa.gov/gistemp/graphs/graph_data/Global_Mean_Estimates_based_on_Land_Data_only/graph.csv

## Supplementary Material

Section A. Experiment 1: A heatmap for independents

FIGURE S1 A heatmap showing the average duration of dwell time on the temperature curve for independents (*N* = 14) in the temperature condition. Participants whose ratings on the political orientation scale were 0 were grouped as independents. Warmer colors represent higher average duration of dwell time.

Section B. Experiment 2: A heatmap for independents

FIGURE S2 A heatmap showing the distribution of the average density of mouse location on the graph for independents (*N* = 11). Participants whose ratings on the political orientation scale were 0 were grouped as liberals and whose ratings on the political orientation scale were above 0 were grouped as conservatives. Warmer colors represent higher average density of mouse location.

## References

[ref1] Bail C. A. Argyle L. P. Brown T. W. Bumpus J. P. Chen H. Hunzaker M. F.. (2018). Exposure to opposing views on social media can increase political polarization. Proc. Natl. Acad. Sci. USA 115, 9216–9221. 10.1073/pnas.1804840115, PMID: 30154168 PMC6140520

[ref2] Bain P. G. Hornsey M. J. Bongiorno R. Jeffries C. (2012). Promoting pro-environmental action in climate change deniers. Nat. Clim. Chang. 2, 600–603. 10.1038/nclimate1532

[ref3] Bosetti V. Weber E. Berger L. Budescu D. V. Liu N. Tavoni M. (2017). COP21 climate negotiators/'responses to climate model forecasts. Nat. Clim. Chang. 7, 185–191. 10.1038/nclimate3208

[ref4] Brenan M. Saad L. (2018). Global warming concern steady despite some partisan shifts. Available at: https://news.gallup.com/poll/231530/global-warming-concern-steady-despite-partisan-shifts.aspx (Accessed November 30, 2018).

[ref5] Cook J. Nuccitelli D. Green S. A. Richardson M. Winkler B. Painting R. (2013). Quantifying the consensus on anthropogenic global warming in the scientific literature. Environ. Res. Lett. 8:024024. 10.1088/1748-9326/8/2/024024

[ref6] Cook J. Oreskes N. Doran P. T. Anderegg W. R. Verheggen B. Maibach E. W. (2016). Consensus on consensus: a synthesis of consensus estimates on human-caused global warming. Environ. Res. Lett. 11:048002. 10.1088/1748-9326/11/4/048002

[ref7] Dale V. H. Joyce L. A. McNulty S. Neilson R. P. Ayres M. P. Flannigan M. D. (2001). Climate change and forest disturbances. Bioscience 51, 723–734. 10.1641/0006-3568(2001)051[0723:CCAFD]2.0.CO;2

[ref8] Drummond C. Fischhoff B. (2017). Individuals with greater science literacy and education have more polarized beliefs on controversial science topics. Proc. Natl. Acad. Sci. USA 114, 9587–9592. 10.1073/pnas.1704882114, PMID: 28827344 PMC5594657

[ref9] Ehret P. J. Boven L. V. Sherman D. K. (2018). Partisan barriers to bipartisanship: understanding climate policy polarization. Soc. Psychol. Personal. Sci. 9, 308–318. 10.1177/1948550618758709

[ref10] Feldman L. Maibach E. W. Roser-Renouf C. Leiserowitz A. (2012). Climate on cable: the nature and impact of global warming coverage on fox news, CNN, and MSNBC. Int. J. Press/Politics 17, 3–31. 10.1177/1940161211425410

[ref11] Festinger L. (1962). A theory of cognitive dissonance (vol. 2). (Redwoodcity, CA: Stanford University Press).

[ref12] Harold J. Lorenzoni I. Shipley T. F. Coventry K. R. (2016). Cognitive and psychological science insights to improve climate change data visualization. Nat. Clim. Chang. 6, 1080–1089. 10.1038/nclimate3162

[ref13] Hastorf A. H. Cantril H. (1954). They saw a game; a case study. J. Abnorm. Soc. Psychol. 49, 129–134. 10.1037/h0057880, PMID: 13128974

[ref14] Hoegh-Guldberg O. Bruno J. F. (2010). The impact of climate change on the world’s marine ecosystems. Science 328, 1523–1528. 10.1126/science.1189930, PMID: 20558709

[ref15] Hornsey M. J. Harris E. A. Bain P. G. Fielding K. S. (2016). Meta-analyses of the determinants and outcomes of belief in climate change. Nat. Clim. Chang. 6, 622–626. 10.1038/nclimate2943

[ref16] Hsiang S. Kopp R. Jina A. Rising J. Delgado M. Mohan S.. (2017). Estimating economic damage from climate change in the United States. Science 356, 1362–1369. 10.1126/science.aal4369, PMID: 28663496

[ref17] Hulme M. (2009). Why we disagree about climate change: Understanding controversy, inaction and opportunity. (Cambridge, UK: Cambridge University Press).

[ref18] Intergovernmental Panel on Climate Change (2014). Summary for policymakers. (Cambridge, UK: Cambridge University Press).

[ref19] Intergovernmental Panel on Climate Change (2015). Climate change 2014: Mitigation of climate change. (Cambridge, UK: Cambridge University Press).

[ref20] Intergovernmental Panel on Climate Change (2018). Summary for policymakers. (Cambridge, UK: Cambridge University Press).

[ref22] Kahan D. (2012). Why we are poles apart on climate change. Nat. News 488:255. 10.1038/488255a, PMID: 22895298

[ref23] Kahan D. M. Jenkins-Smith H. Braman D. (2011). Cultural cognition of scientific consensus. J. Risk Res. 14, 147–174. 10.1080/13669877.2010.511246

[ref24] Kahan D. M. Peters E. Dawson E. C. Slovic P. (2017). Motivated numeracy and enlightened self-government. Behav. Public Policy 1, 54–86. 10.1017/bpp.2016.2

[ref25] Kim N. W. Bylinskii Z. Borkin M. A. Gajos K. Z. Oliva A. Durand F. (2017). BubbleView: an interface for crowdsourcing image importance maps and tracking visual attention. ACM Trans. Comput.-Hum. Interact. 24, 36:1–36:40. 10.1145/3131275

[ref26] Kniveton D. (2017). Sea-level-rise impacts: questioning inevitable migration. Nat. Clim. Chang. 7, 548–549. 10.1038/nclimate3346

[ref27] Leiserowitz A. (2006). Climate change risk perception and policy preferences: the role of affect, imagery, and values. Clim. Chang. 77, 45–72. 10.1007/s10584-006-9059-9

[ref28] Levitus S. Antonov J. Boyer T. Baranova O. Garcia H. Locarnini R. (2017). NCEI ocean heat content, temperature anomalies, salinity anomalies, thermosteric sea level anomalies, halosteric sea level anomalies, and total steric sea level anomalies from 1955 to present calculated from in situ oceanographic subsurface profile data (NCEI Accession 0164586). Version 4.4. NOAA National Centers for Environmental Information. Dataset.

[ref29] Lobell D. B. Burke M. B. Tebaldi C. Mastrandrea M. D. Falcon W. P. Naylor R. L. (2008). Prioritizing climate change adaptation needs for food security in 2030. Science 319, 607–610. 10.1126/science.1152339, PMID: 18239122

[ref30] Lorenzoni I. Nicholson-Cole S. Whitmarsh L. (2007). Barriers perceived to engaging with climate change among the UK public and their policy implications. Glob. Environ. Chang. 17, 445–459. 10.1016/j.gloenvcha.2007.01.004

[ref31] Lorenzoni I. Pidgeon N. F. (2006). Public views on climate change: European and USA perspectives. Clim. Chang. 77, 73–95. 10.1007/s10584-006-9072-z

[ref32] McCright A. M. Dunlap R. E. (2011). The politicization of climate change and polarization in the American public’s views of global warming, 2001–2010. Sociol. Q. 52, 155–194. 10.1111/j.1533-8525.2011.01198.x

[ref34] National Oceanic and Atmospheric Administration (2018a). Global climate change indicators. Available at: https://www.ncdc.noaa.gov/monitoring-references/faq/indicators.php (Accessed October 13, 2018).

[ref35] National Oceanic and Atmospheric Administration (2018b). Trends in atmospheric carbon dioxide. Available at: https://www.esrl.noaa.gov/gmd/ccgg/trends/global.html (Accessed October 13, 2018).

[ref36] Nerem R. S. Beckley B. D. Fasullo J. T. Hamlington B. D. Masters D. Mitchum G. T. (2018). Climate-change–driven accelerated sea-level rise detected in the altimeter era. Proc. Natl. Acad. Sci. USA 115, 2022–2025. 10.1073/pnas.1717312115, PMID: 29440401 PMC5834701

[ref37] Newman T. P. Nisbet E. C. Nisbet M. C. (2018). Climate change, cultural cognition, and media effects: worldviews drive news selectivity, biased processing, and polarized attitudes. Public Underst. Sci. 27, 985–1002. 10.1177/0963662518801170, PMID: 30253695

[ref38] Nickerson R. S. (1998). Confirmation bias: a ubiquitous phenomenon in many guises. Rev. Gen. Psychol. 2, 175–220. 10.1037/1089-2680.2.2.175

[ref39] Norton A. Leaman J. (2004). The day after tomorrow: Public opinion on climate change. (London: MORI Social Research Institute).

[ref40] Petit J. R. Jouzel J. Raynaud D. Barkov N. I. Barnola J. M. Basile I. (1999). Climate and atmospheric history of the past 420,000 years from the Vostok ice core, Antarctica. Nature 399, 429–436. 10.1038/20859

[ref41] Petty R. E. Cacioppo J. T. (1986). Communication and persuasion: Central and peripheral routes to attitude change. (New York: Springer-Verlag).

[ref42] Pew Research Center (2017). Global warming and environmental regulation, personal environmentalism. Available at: http://www.people-press.org/2017/10/05/7-global-warming-and-environmental-regulation-personal-environmentalism/ (Accessed November 30, 2018).

[ref43] Pew Research Center (2018). Economic issues decline among public’s policy priorities. Available at: http://www.people-press.org/2018/01/25/economic-issues-decline-among-publics-policy-priorities/ (Accessed November 30, 2018).

[ref44] Poortinga W. Spence A. Whitmarsh L. Capstick S. Pidgeon N. F. (2011). Uncertain climate: an investigation into public scepticism about anthropogenic climate change. Glob. Environ. Chang. 21, 1015–1024. 10.1016/j.gloenvcha.2011.03.001

[ref45] Robinson D. A. Hall D. K. Mote T. L. (2014). MEaSUREs Northern Hemisphere Terrestrial Snow Cover Extent Daily 25km EASE-Grid 2.0, Version 1. Boulder, Colorado, USA: NASA National Snow and Ice Data Center Distributed Active Archive Center.

[ref46] Rosenzweig C. Karoly D. Vicarelli M. Neofotis P. Wu Q. Casassa G.. (2008). Attributing physical and biological impacts to anthropogenic climate change. Nature 453, 353–357. 10.1038/nature06937, PMID: 18480817

[ref47] Seidl R. Thom D. Kautz M. Martin-Benito D. Peltoniemi M. Vacchiano G.. (2017). Forest disturbances under climate change. Nat. Clim. Chang. 7, 395–402. 10.1038/nclimate3303, PMID: 28861124 PMC5572641

[ref48] Shi J. Visschers V. H. Siegrist M. Arvai J. (2016). Knowledge as a driver of public perceptions about climate change reassessed. Nat. Clim. Chang. 6, 759–762. 10.1038/nclimate2997

[ref49] Van Boven L. Ehret P. J. Sherman D. K. (2018). Psychological barriers to bipartisan public support for climate policy. Perspect. Psychol. Sci. 13, 492–507. 10.1177/1745691617748966, PMID: 29961412

[ref50] von Borgstede C. Andersson M. Hansla A. (2014). Value-congruent information processing: the role of issue involvement and argument strength. Basic Appl. Soc. Psychol. 36, 461–477. 10.1080/01973533.2014.958226

[ref51] Weber E. U. Stern P. C. (2011). Public understanding of climate change in the United States. Am. Psychol. 66:315–328. 10.1037/a0023253, PMID: 21553956

[ref52] Whitman J. C. Zhao J. Roberts K. H. Todd R. M. (2018). Political orientation and climate concern shape visual attention to climate change. Clim. Chang. 147, 383–394. 10.1007/s10584-018-2147-9

[ref53] Wuebbles D. J. Fahey D. W. Hibbard K. A. DeAngelo B. Doherty S. Hayhoe K. (2017). “Executive summary” in Climate science special report: Fourth national climate assessment, volume I eds. Wuebbles D. J. Fahey D. W. Hibbard K. A. Dokken D. J. Stewart B. C. Maycock T. K. (Washington, DC, USA: U.S. Global Change Research Program), 12–34. 10.7930/J0DJ5CTG

[ref54] Zhao J. (2017). Influencing policymakers. Nat. Clim. Chang. 7, 173–174. 10.1038/nclimate3215

