# Supplementary figures and images for: Motivated Attention in Climate Change Perception and Action
Source: Front Psychol. 2019 Jul 16;10:1541. doi: 10.3389/fpsyg.2019.01541 (PMC6660247; doi:10.3389/fpsyg.2019.01541)

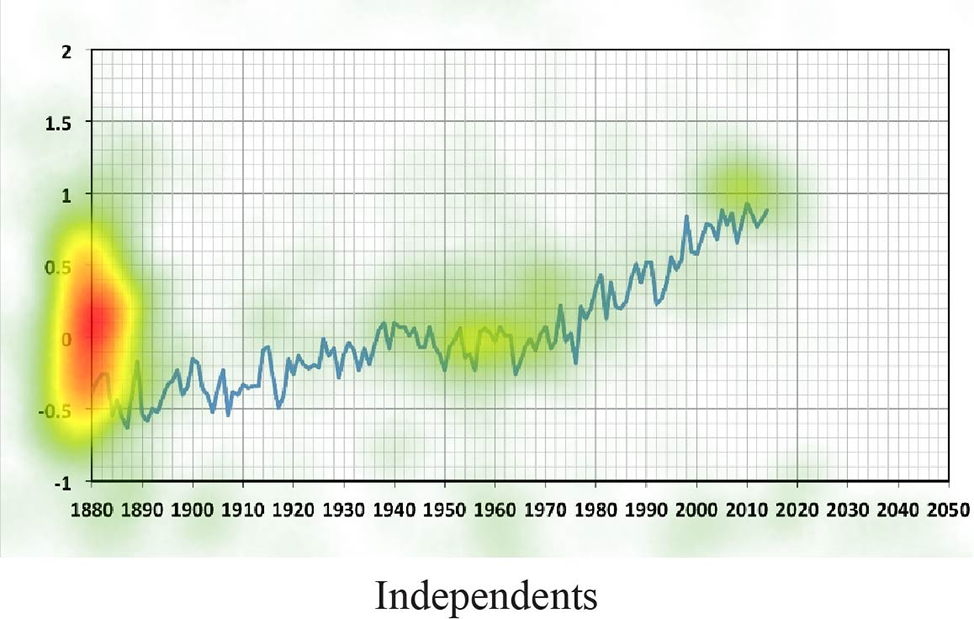

Supplement: FIGURE S1 — A heatmap showing the average duration of dwell time on the temperature curve for independents (N = 14) in the temperature condition. Participants whose ratings on the political orientation scale were 0 were grouped as independents. Warmer colors represent higher average duration of dwell time. [file Image_1.tif]

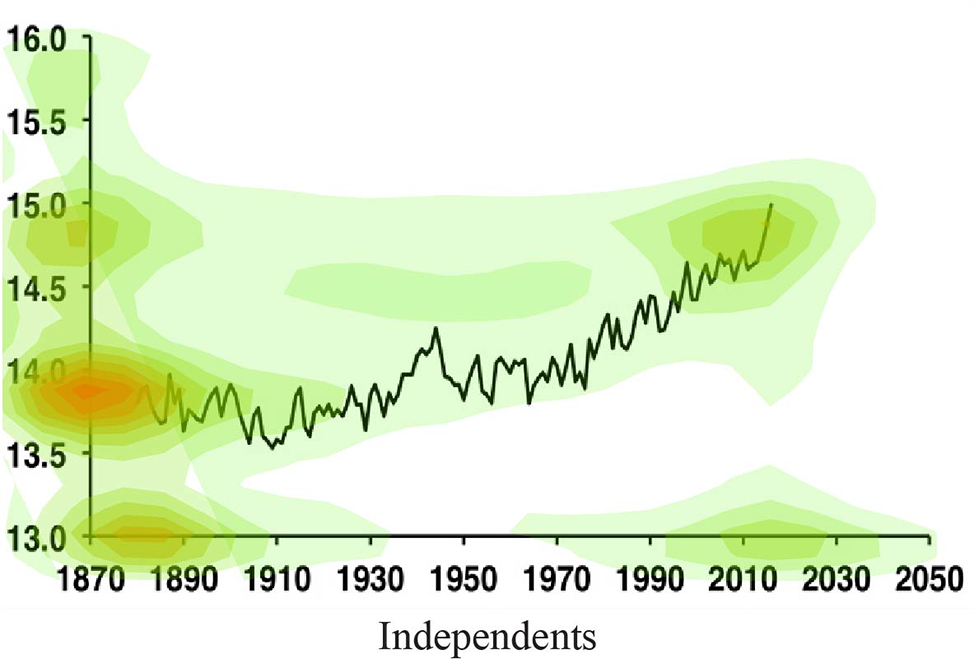

Supplement: FIGURE S2 — A heatmap showing the distribution of the average density of mouse location on the graph for independents (N = 11). Participants whose ratings on the political orientation scale were 0 were grouped as liberals and whose ratings on the political orientation scale were above 0 were grouped as conservatives. Warmer colors represent higher average density of mouse location. [file Image_2.tif]
